# Monitoring protein phosphorylation by acrylamide pendant Phos-Tag™ in various plants

**DOI:** 10.3389/fpls.2015.00336

**Published:** 2015-05-13

**Authors:** Slávka Bekešová, George Komis, Pavel Křenek, Petra Vyplelová, Miroslav Ovečka, Ivan Luptovčiak, Peter Illés, Anna Kuchařová, Jozef Šamaj

**Affiliations:** Department of Cell Biology, Centre of the Region Haná for Biotechnological and Agricultural Research, Palacký University OlomoucOlomouc, Czech Republic

**Keywords:** protein phosphorylation, SDS-PAGE Phos-Tag^TM^, *Arabidopsis thaliana*, *Hordeum vulgare*, *Medicago sativa*, *Triticum turgidum*, mitogen activated protein kinase

## Abstract

The aim of the present study is to rationalize acrylamide pendant Phos-Tag™ in-gel discrimination of phosphorylated and non-phosphorylated plant protein species with standard immunoblot analysis, and optimize sample preparation, efficient electrophoretic separation and transfer. We tested variants of the method including extraction buffers suitable for preservation of phosphorylated protein species in crude extracts from plants and we addressed the importance of the cation (Mn^2+^ or Zn^2+^) used in the gel recipe for efficient transfer to PVDF membranes for further immunoblot analysis. We demonstrate the monitoring of *Medicago sativa* stress-induced mitogen activated protein kinase (SIMK) in stress-treated wild type plants and transgenic *SIMKK RNAi* line. We further show the hyperosmotically-induced phosphorylation of the previously uncharacterized HvMPK4 of barley. The method is validated using inducible phosphorylation of barley and wheat α-tubulin and of Arabidopsis MPK6. Acrylamide pendant Phos-Tag™offers a flexible tool for studying protein phosphorylation in crops and Arabidopsis circumventing radioactive labeling and the use of phosphorylation specific antibodies.

## Introduction

Protein post-translational modifications (PTMs) and particularly reversible protein phosphorylation are versatile switches regulating protein structure, interactions, function, and subcellular localization (Müller et al., 2010; Nishi et al., 2013, 2014; Offringa and Huang, 2013; Smékalová et al., 2014) under conditional signaling (Ovečka et al., 2014) or during developmental processes (Beck et al., 2010; Smékalová et al., 2014). The inducible phosphorylation of few or even a single protein species might have global consequences on cell physiology, if these proteins—e.g., transcription factors—are involved in central processes such as transactivation of gene expression (Ishihama and Yoshioka, 2012; Meng et al., 2013). Protein phosphorylation is catalyzed in a targeted manner by a large family of enzymes called protein kinases (Dissmeyer and Schnittger, 2011). Protein kinases are attracted by protein substrates via specific and complementary motifs present on both interaction partners as thoroughly studied, for example, in the case of mitogen activated protein kinases (MAPKs; Salazar and Höfer, 2009; Šamajová et al., 2013; Pitzschke, 2015).

The efficiency, the complexity, the spatiotemporal coordination and finally the physiological importance of reversible protein phosphorylation nourished the development of quantitative experimental procedures to address site-specificity, kinetics, and functional consequences of protein phosphorylation (Liu and Chance, 2014).

At large scale, phosphopeptides may be identified by mass spectrometric analyses following appropriate phosphopeptide enrichment strategies (St-Denis and Gingras, 2012) and by subtractive motif- or drug-based approaches, while global phosphoproteomic analyses may provide information on kinase-specific phosphoproteome landscapes (Ruse et al., 2008; Boesger et al., 2012).

Small scale approaches incorporating radioactive metabolic labeling followed by in gel (Cuenda, 2000) or in solution (Dickson, 2008) assays, may provide highly sensitive quantitative and kinetic monitoring of either kinase activity, or the phosphorylation process of a kinase substrate. However, such approaches mandate severe safety precautions, specific training of personnel, and cost-demanding laboratory setup, while inevitably producing undesirable radioactive waste. In addition, these are *in vitro* assays, presenting the potential of protein phosphorylation but not the actual phosphorylation state within the cell.

Protein phosphorylation in a small scale may be also addressed by phosphorylation-specific antibodies raised against either the protein of interest, or more generally against phosphorylated aminoacids. In the latter case, the lack of specificity of antibodies can be a problem, since phospho-tyrosine monoclonal antibodies have adequate affinity for phosphorylated Tyr residues, but monoclonal antibodies against phospho-serine or phospho-threonine are unpopular because their affinities and specificities are not optimal. An excellent non-radioactive alternative is the separation of protein species by means of one dimensional isoelectric focusing (1D IEF) which can be then coupled with western blot analysis of the proteins of interest in *in vivo* assays, representing the status quo of the cell (Anderson and Peck, 2008, 2014).

Herein we use an approach somewhat less demanding than 1D IEF in terms of both equipment and reagents since it can be carried out using standard SDS PAGE minigel setup. This method relies on the differential electrophoretic migration of phosphoprotein species compared to their non-phosphorylated counterparts by means of complexing protein phosphogroups with transition metal cations immobilized in-gel using a covalently incorporated chelating agent trademarked as Phos-Tag™(Kinoshita et al., 2006; Kinoshita and Kinoshita-Kikuta, 2011; Kinoshita-Kikuta et al., 2014).

We provide a thorough protocol for acrylamide pendant Phos-Tag™ separation of phosphorylated and non-phosphorylated proteins in four plant species (three crops; *Medicago sativa*, alfalfa; *Hordeum vulgare*, barley; and *Triticum turgidum*, wheat and one genetic model; *Arabidopsis thaliana*). This methodology aims to identify single or multiple phosphorylation events of proteins of interest, provided that specific antibodies for the protein under study are available. The method requires prior knowledge that the protein of interest is or might be conditionally phosphorylated but it cannot identify the phosphorylated aminoacid *per se*. This problem can be circumvented if prior to electrophoretic separation the protein is selectively immunoprecipitated and subsequently probed with anti-phospho-Ser/Thr or Tyr antibodies. Importantly, Phos-Tag™ protocols have been developed for monitoring aspartate phosphorylation (Barbieri and Stock, 2008), thiophosphorylation (Kinoshita et al., 2014), and histidine phosphorylation (Ishii et al., 2013). In the present protocol examples of protein phosphorylation elucidated by acrylamide pendant Phos-Tag™ and subsequent western blot analysis, include the *Medicago sativa* MAPK called SIMK (stress-induced MAPK homologous to *At*MPK6 probed with the same antibody as *At*MPK6), *Arabidopsis thaliana* MAPKs *At*MPK3 and *At*MPK6, *Hordeum vulgare* MAPK *Hv*MPK4 and the structural protein alpha tubulin in *Hordeum vulgare* and *Triticum turgidum* as it was recently identified as a target of hyperosmotically induced protein phosphorylation in rice and *Arabidopsis thaliana* (Ban et al., 2013; Fujita et al., 2013). The protocol is adapted to crude protein extracts from diverse plant material. Our laboratory routinely employs Phos-Tag™ technology to decipher phosphorylation and activation of MAPKs (Beck et al., 2010) or MAPK substrates (Panteris et al., 2010; Smékalová et al., 2014) in the model plant *Arabidopsis thaliana*. Importantly we employed a transgenic line of alfalfa with silenced SIMKK (*SIMKK RNAi*) which is the upstream regulator of SIMK and also studied the hyperosmotic activation of the barley ortholog of MPK4 by means of a custom raised monospecific antibody.

## Materials and methods

### Chemicals

All common chemicals were of defined purity (pro analysis) and purchased from Sigma (www.sigma-aldrich.com) and Duchefa (www.duchefa-biochemie.com). Phos-Tag™ was from Wako Pure Chemical Industries Ltd (www.wako-chem.co.jp). Protease and phosphatase inhibitor cocktails (Complete™, EDTA-free, and PhosStop™) were from Roche (lifescience.roche.com). λ-Phosphatase was from New England Biolabs (www.neb.com). The following antibodies were used: anti-AtMPK6 affinity purified rabbit polyclonal IgGs (Sigma), anti-pTEpY rabbit polyclonal IgGs (Cell Signaling; www.cellsignal.com), anti-HvMPK4 rabbit polyclonal IgGs (designated as JK4 raised against the peptide 288-PRQDFRLRFRNM-299 derived from the predicted sequence in Genbank under the accession number AK366765; custom produced by Genscript, www.genscript.com), mouse monoclonal anti-alpha tubulin (clone DM1a; Abcam; www.abcam.com) and horseradish peroxidase conjugated goat anti-rabbit IgGs and rabbit anti-mouse IgGs (both from Santa Cruz Biotech; www.scbt.com). Polyclonal serum against MPK6 was raised against the carboxylterminal residues 384–395 of *Arabidopsis thaliana* MPK6 and was peptide affinity purified. The anti-pTEpY polyclonal serum was generated using a peptide surrounding the respective motif of mammalian ERK1/2 (extracellular signal related protein kinase 1 and 2). The anti-pTEpY polyclonal serum was affinity purified against the phospho-peptide used for immunization and against the same peptide in its unphosphorylated form. Monoclonal anti-α tubulin antibody, clone DM1a, was generated from hybridomas of mice injected with chick brain tubulin and corresponds to the C-terminus of α tubulin (residues 426–430). The peptide used for JK4 production was synthesized, coupled to keyhole limpet hemocyanin and injected to New Zealand rabbits according to the protocol of the producer. Bradford reagent, acrylamide/bis-acrylamide mixture (30% w/v solution, 37.5:1 ratio acrylamide to bis-acrylamide), pre-stained molecular ladder and enhanced chemiluminescence (ECL; Clarity™) reagent were all from Bio-Rad (www.bio-rad.com).

### Plant material and treatments

In the present study we used seedlings of *Medicago sativa* cv. Regen SY (RSY), *Hordeum vulgare* cv. Golden Promise, *Triticum turgidum* cv. Athos and *Arabidopsis thaliana* ecotype Columbia (Col-0). Before plating, seeds of *Arabidopsis thaliana* and *Medicago sativa* and caryopses of *Hordeum vulgare* and *Triticum turgidum*, were appropriately surface sterilized first with 70% v/v aqueous ethanol for 2 min and subsequently with 10% v/v aqueous bleach solution supplemented with 0.01% v/v Tween-20 for 10 min. Following washing with sterile, distilled water, all plant material was plated to ½Murashige Skoog nutrient medium solidified with 0.8% w/v Phytagel™ and allowed to grow in controlled environmental chambers with standard light:dark cycle (16:8 h)/ temperature (21°C day/18°C night)/ humidity (60% day/70% night) settings. For treatments, 10 day old *Arabidopsis thaliana* seedlings were treated with 15 mM H_2_O_2_ in liquid ½MS for 30 min. 3–4 day old seedlings of *Hordeum vulgare* and *Triticum turgidum* were treated with 0.8 M sorbitol in liquid ½MS for 30 min. Wild type seedlings of *Medicago sativa* were treated with 250 mM NaCl, 15 mM H_2_O_2_, and 0.8 M sorbitol in liquid ½MS for 30 min and RNAi expressing *Medicago sativa* plants growing in soil pots were treated by wounding by lightly scoring leaves with a sharp razor blade and collected in liquid nitrogen 5 min post-wounding.

### Cloning of *SIMKK RNAi* and transformation of *M. sativa*

To obtain *SIMKK RNAi* line of *M. sativa*, leaves of mature plants were transformed with *A. tumefaciens* carrying pHellsgate12-SIMKKi expression plasmid. Construction of pHellsgate12-SIMKKi expression plasmid was performed by Gateway® recombination cloning, using pDONR™207 (Life Technologies) donor vector and pHellsgate12 destination vector (obtained from CSIRO Plant Industry, Australia). In the first step, 366 bp PCR fragment was synthesized using SIMKKiFor primer (GGGGACAAGTTTGTACAAAAAAGCAGGCTTCTTAAGGATATATGGAGTTTAGGCGTGAG), SIMKKiRev primer (GGGGACCACTTTGTACAAGAAAGCTGGGTATCTTGGTGGTGGAGGAAGTAAC) and total cDNA of *M. sativa* as template. Primers were designed according to standards for Gateway® cloning. In addition, restriction site for AflIII (CTTAAG) was inserted between *att*B1 site and template-complementary site of forward primer, enabling further verification of correct orientation of fragments in expression vector. Consequently, BP recombination reaction (recombination between attB and attP sites) was performed to obtain pDONR207-SIMKKi entry vector by cloning of synthesized PCR fragment into pDONR™207 vector, using Gateway® BP Clonase® II Enzyme Mix (Life Technologies) according to standard protocol of manufacturer. Construction of final expression plasmid pHellsgate12-SIMKKi was performed by recombination of prepared pDONR207-SIMKKi entry vector and pHellsgate12 destination vector (LR reaction; recombination between attL and attR sites), using Gateway® LR Clonase® II Enzyme Mix (Life Technologies) following protocol of manufacturer.

*Agrobacterium tumefaciens* strain GV3101::pMP90 was transformed with *SIMKK-RNAi* construct and used for stable transformation of *Medicago sativa*. Young leaves from *in vitro* growing plants of the cultivar RSY were selected as explant source. The transformation was performed by cocultivation method (Samac and Austin-Phillips, 2006). Induction of callogenesis from leaf explants, production of somatic embryos from calli, development of shoots and their rooting were performed under selective conditions. Regenerated plants (Austin et al., 1995) that grew on media with selective kanamycin marker were further tested by genotyping. To confirm the presence of the construct in transgenic *Medicago* plants, two sets of primers for verification of presence of the hairpin were tested. Genomic DNA from control non transformed regenerated plants and SIMKK-RNAi-transformed plants was isolated using CTAB method (Semagn, 2014). Primers were tested and after optimization of PCR conditions, all of them were functional.

### Assessment of transcripts in *M. sativa SIMKK RNAi* line by RT-qPCr

Transcript levels of *SIMKK* in the *RNAi* and the control *Medicago sativa* lines were quantified by RT qPCR of total RNA isolated from respective seedlings with Trizol® reagent (Life Technologies, www.lifetechnologies.com) using the following primer sets for SIMKK: CACCAGAAGCTCCAACGACTG (forward) and CGACGGGTCTCTCTGCAAAC (reverse), and the product was normalized against *ACTIN2* transcript levels (GGATAAGAGGTGAGATCGGAGGG; forward and GCAACCAACCTACAGACATCCAG; reverse).

### Protein extraction and λ-phosphatase treatment

In all cases plant material was harvested immediately before protein isolation and was flash frozen in liquid nitrogen. Material was then crashed into fine powder, collected in prechilled and preweighted microfuge tubes and stored under liquid nitrogen until further processing. Protein extracts were prepared with the following extraction solutions:

RIPA (radio-immunoprecipitation assay) buffer: 25 mM Tris-HCl (pH 7.6), 150 mM NaCl, 1% v/v Triton X-100, 0.5% w/v sodium deoxycholate, 0.1% w/v SDS.

Buffer E: 50 mM HEPES (pH 7.5), 75 mM NaCl, 1 mM EGTA, 1 mM MgCl_2_, 1 mM NaF, 10% v/v glycerol.

Buffer B: 20 mM Na-phosphate solution (pH 7.4) (mixture of 2 ml of NaH_2_PO_4_ with 8 ml Na_2_HPO_4_ from 200 mM stock solutions), 50 mM ß-glycerophosphate, 100 μM Na_3_VO_4_.

All mentioned buffers were appropriately supplemented with Complete™ EDTA-free and PhosSTOP™.

For the extraction, liquid nitrogen powders of plant material were extracted on ice for 30 min with 1–2 volumes of appropriate extraction buffer. RIPA was the buffer of choice for *Arabidopsis thaliana* seedlings, buffer E and buffer B worked equally well for *Medicago sativa* and buffer B was exclusively used for extracts of *Hordeum vulgare* and *Triticum turgidum* as mentioned by Ban et al. (2013). Subsequently extracts were cleared at 23000× g, 10 min, 2°C and the resulting supernate was used as crude protein extract. Protein content in the original extract was quantified by Bradford assay and it was further cleaned by repeated rounds of quantitative cold acetone precipitation (80% v/v; Komis et al., 2014). By acetone precipitation proteins are selectively precipitated leaving salts and other soluble impurities in solution. This is quite critical since even minimal amounts of phosphates, especially in the case of buffer B, will interfere with the assay.

In some instances phosphorylation discriminated in western blots following acrylamide pendant Phos-Tag™ SDS-PAGE was verified by treatment of protein extracts with λ PPase. In this case, protein extracts corresponding to a total of 100 μg were successively precipitated with 80% v/v acetone (at least 3 times) at −20°C for at least 1 h each time in order to remove phosphatase inhibitor contaminants from the protein mixture. The final protein pellet was briefly dried and finally resuspended to λ PPase reaction buffer (50 mM HEPES pH 7.5, 10 mM NaCl, 2 mM DTT, 0.01% (v/v) Brij® 35 (supplied prepared in λ PPase kit of New England Biolabs). The reaction mixture was then supplemented with 200–400 units of λ PPase and the reaction was allowed to proceed for 30 min at 30°C according to manufacturer's instructions. At the end of the reaction, the product was precipitated by adding 4 volumes of acetone and kept overnight at −20°C. The resulting pellet was precipitated at 23000× g, 10 min, and 2°C and allowed to slightly dry before dissolving in 1× Laemmli sample buffer and heat denature accordingly.

### Electrophoresis and western blot

Two different electrophoresis procedures were followed. One involving the in-gel combination of Phos-Tag™ with Mn^2+^ and the other involving the combination of Phos-Tag™ with Zn^2+^. For the Mn^2+^/Phos-tag™ combination the standard Tris-Cl buffered stacking (4% w/v acrylamide) and separating (8% w/v acrylamide) gel recipes were applied. The Zn^2+^/Phos-tag™ stacking and separating gels were prepared as follows:

Stacking Zn^2+^/Phos-tag™ gel buffer: 357 mM Bis-Tris (pH 6.8), 0.1% v/v TEMED, 0.05% w/v ammonium persulfate (APS). Acrylamide, bis-acrylamide and water should be adjusted according to the desired gel pore size. In the present protocol a 4% w/v acrylamide stacking gel was used.

Separating Zn^2+^/Phos-tag™ gel buffer: 357 mM Bis-Tris (pH 6.8), 25 μM Phos-Tag™, 50 μM Zn(NO_3_)_2_, 0.05% v/v TEMED, 0.025% w/v APS. Acrylamide, bis-acrylamide and water should be adjusted according to the desired gel pore size. In the present protocol an 8% w/v acrylamide separating gel was used. The concentration of Phost-Tag™, will impact the separation and the transfer efficiency of the gel. For higher MW proteins that are already difficult to transfer, lower Phos-Tag™ concentrations (e.g., 5 μM) have been used quite efficiently (Kinoshita et al., 2009).

The running buffer for standard and Mn^2+^/ Phos-Tag™ gels was 25 mM Tris base, 192 mM glycine and 0.1% w/v SDS. For Zn^2+^/ Phos-Tag™ the running buffer consisted of 100 mM Tris, 100 mM MOPS, and 0.1% (w/v) SDS to which 5 mM of sodium bisulfite were added immediately before electrophoresis.

In all cases, the power supply settings were adjusted for optimal separation of phosphorylated and non-phosphorylated protein species. For this reason, all gels run at 10–15 mA/gel constant current and electrophoresis required 4–6 h for completion.

For transfer of gel separated proteins to PVDF membranes for subsequent immunoblotting of the protein of interest, gels were pretreated with washing in methanol-free transfer buffer (25 mM Tris-Cl pH 7.4, 192 mM glycine, 10 mM EDTA) for 6 times 10 min each in order to remove bivalent cations that would immobilize phosphorylated proteins in the gel not allowing their transfer to PVDF membrane. Finally the gel was equilibrated for 2 × 10 min in methanol-free transfer buffer with 1 mM EDTA. Gels were transferred to PVDF membranes with transfer buffer containing 5% v/v methanol and 1 mM EDTA at 24V overnight. For efficient transfer of Phos-Tag™ gel separated proteins we used wet-tank transfer approach since semi-dry transfer protocols are less efficient and complicate the procedure (Kosako, 2009).

Following electrophoretic transfer, membranes were allowed to completely dry. Subsequently they were reactivated in methanol and immediately stained with 3% w/v Ponceau S in 5% v/v aqueous solution of acetic acid to validate transfer efficiency. Subsequently membranes were thoroughly destained with milliQ water and TBST (10 mM Tris–HCl (pH 7.5), 100 mM NaCl, and 0.10% v/v Tween-20). Non-specific antibody binding was blocked by incubating membranes in 6% w/v BSA in TBST for at least 1 h. Primary antibodies were appropriately diluted in 6% w/v BSA in TBST and incubation of the membranes was carried out overnight at 4°C. Following extensive washing in TBST (6 × 10 min), membranes were incubated with diluted HRP-conjugated secondary antibodies for 1 h. After washing in TBST (6 × 10 min) membranes were incubated with ECL reagent according to manufacturer's instructions. Immunoreactive bands were documented with a BioRad ChemiDoc™ MP System. Western blots of Phos-Tag™ gels cannot be used to make MW assignments. Prestained MW markers migrate anomalously in such gels, whereas all proteins migrate slower compared to standard SDS-PAGE gels. Therefore, protein identity should be based on known immunoreactivity.

## Results

As already mentioned Phos-Tag™ identification of phosphorylated proteins following SDS-PAGE separation and western blotting has been well-established in *Arabidopsis thaliana* (Bethke et al., 2009; Beck et al., 2010; Mao et al., 2011; Fujita et al., 2013; Li et al., 2014; Smékalová et al., 2014; Kang et al., 2015) while few applications included dicotyledonous and monocotyledonous crops (Panteris et al., 2010; Taylor et al., 2012; Ban et al., 2013). Herein we attempt to provide proper conditions for preservation of phosphorylated proteins upon extraction, for their adequate separation in acrylamide pendant Phos-Tag™ SDS-PAGEs and their quantitative transfer to solid membranes for subsequent western blot-based probing of the proteins of interest.

### Salt, oxidative, and wound-induced activation of *M. sativa* SIMK

**Figure 1:**
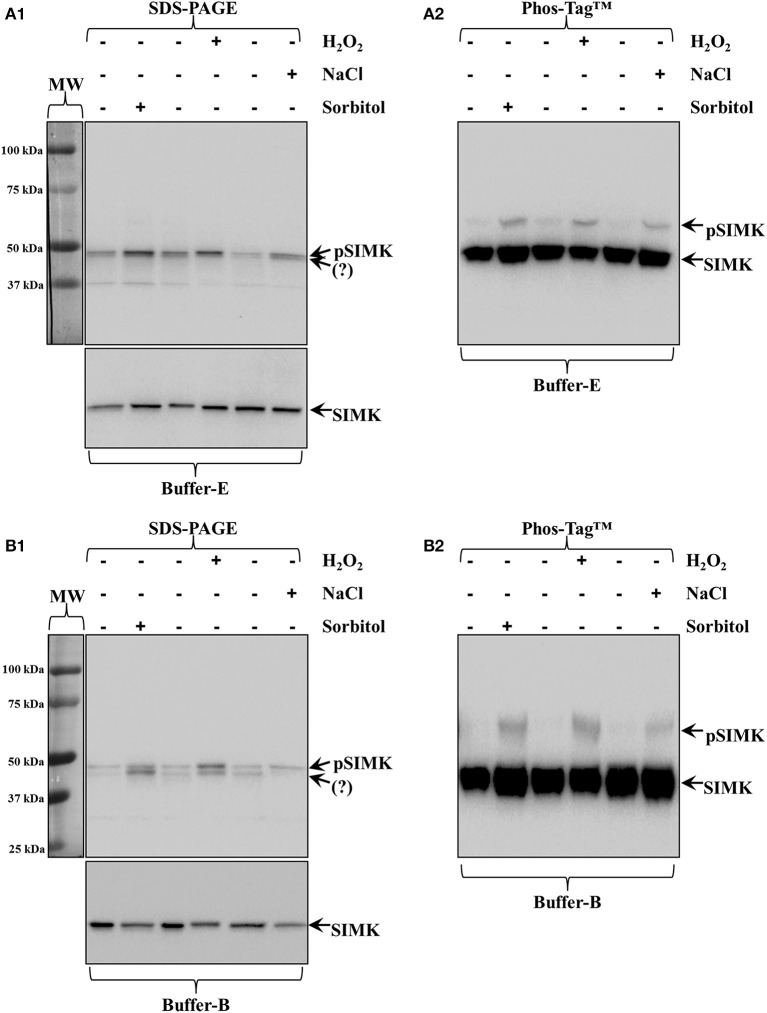
**Activation of** ***Medicago sativa*** **SIMK by three different stresses (250 mM NaCl, 15 mM H_2_O_2_ and 0.8 M sorbitol, all applied for a total of 30 min)**. **(A1,A2)** All stresses induce the accumulation of phosphorylated MAPK species compared to untreated seedlings as recognized by pTEpY antibody in extracts prepared in buffer E **(A1)** which by Phos-Tag™ **(A2)** show clear SIMK activation (top arrow, pSIMK; bottom arrow, SIMK). **(B1,B2)** The same samples extracted with buffer B show better discrimination of pTEpY immunoreactive species in western blots following common SDS-PAGE **(B1)** and much better separation of pSIMK (top arrow) and SIMK (bottom arrow) in Phos-Tag™ gels **(B2)**. Panels on the left of **(A1,B1)** correspond to images of the protein ladder following Ponceau S staining of the membrane. Question marks in **(A1,B1)** denote unknown anti-pTEpY immunoreactive species.

A prominent MAPK of *Medicago sativa* is SIMK which can be equally activated by exposure to hyperosmotic salt or sorbitol solutions or by wounding and oxidative stress (Kiegerl et al., 2000; Cardinale et al., 2002). In all cases the strongest activator of SIMK is the MAPKK called SIMKK which can directly activate SIMK (Cardinale et al., 2002). Moreover, SIMK is a highly conserved homolog of AtMPK6 with absolute identity in the carboxylterminal region. Therefore, a commercial antibody generated against the 10 carboxylterminal residues of AtMPK6 can equally detect SIMK (46 kDa; Šamaj et al., 2002) in appropriate western blots (Ovečka et al., 2014; Figure 1A1 bottom panel; arrow). For this reason we monitored the activation of *Medicago sativa* SIMK following salt, sorbitol and wounding stress in roots of either wild type seedlings or in leaves of wound-inflicted plants stably transformed with a *siRNA SIMKK* construct using this SIMK antibody. Therefore, this SIMKK silenced *M. sativa* line was used for further Phos-tag and western blot analyses.

The activation of SIMK upon brief salt, H_2_O_2_ and sorbitol treatment of *Medicago sativa* roots (see Materials and Methods) was confirmed by western blot analysis of protein extracts acquired in buffer E and buffer B using a commercial anti-pTEpY antibody (Figure 1A1, top panel; arrow and Figure 1B1, top panel, top arrow respectively).

In the case of buffer E extracted proteins, the anti-pTEpY antibody detected one specific band at the expected molecular weight for SIMK (Figure 1A1 top panel, top arrow) and a second band corresponding to another unidentified salt-induced MAPK (Figure 1A1 top panel, bottom arrow). The first band detected with the anti-pTEpY antibody was coinciding with a band detected with anti-SIMK antibody (Figure 1A1 bottom panel, arrow) suggesting that it corresponds to phosphorylated SIMK. The same pattern was revealed when buffer B extracted proteins were similarly separated and immunoblotted, although they showed better separation of the pSIMK band when compared to E buffer extracted proteins (Figure 1B1, top arrow) from the band corresponding to the unidentified salt-induced MAPK (Figure 1B1, bottom arrow) as further corroborated with anti-SIMK antibody (Figure 1B1, bottom panel, arrow).

Extracts in buffer E and buffer B were separated in Zn^2+^/Phos-Tag™ gel and immunoprobed with anti-SIMK antibody resulting in two well discriminated bands in both cases separating unphosphorylated and phosphorylated SIMK (Figures 1A2,B2; bottom arrows, SIMK; top arrows, pSIMK).

**Figure 2:**
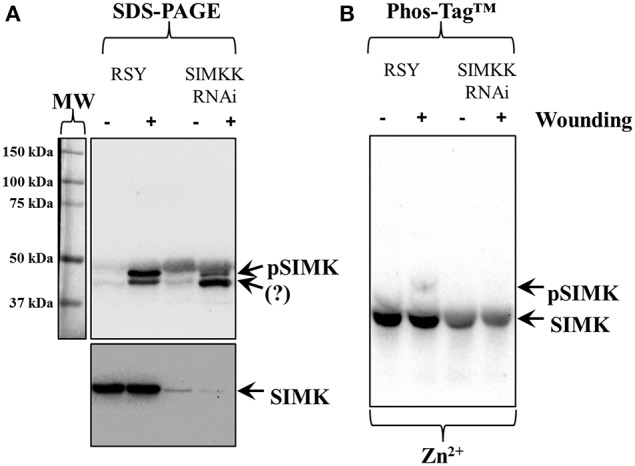
**Effects of silencing of SIMKK on inducible activation and total levels of SIMK in SIMKK RNAi expressing** ***Medicago sativa*** **plants following wound induction. (A)** extracts control (RSY) and RNAi (SIMKK RNAi) obtained from resting or wounded leaves, where probed against anti-pTEpY antibody recognizing phosphorylated SIMK (**A**, top panel, top arrow, pSIMK) or with anti-MPK6 antibody recognizing total levels of SIMK (**A**, bottom panel, arrow, SIMK). The RNAi line shows reduced accumulation of anti-pTEpY immunoreactivity corresponding to SIMK (**A**, top panel, top arrow, pSIMK). Surprisingly the total levels of SIMK where found reduced in both RNAi lines compared to the controls (**A**, bottom panel, arrow, SIMK). **(B)** Identical extracts as those used for Panel **(A)** where separated in Zn^2+^/Phos-Tag™ gel and probed with anti-MPK6 antibody, showing the absence of the upper band corresponding to pSIMK (top arrow) in the RNAi line. The bottom arrow denotes the unphosphorylated form of SIMK. Panel on the left of **(A)** corresponds to image of the protein ladder following Ponceau S staining of the membrane.

Phosphorylation of SIMK was further pursued in wounded *Medicago sativa* leaves from control plants and from plants expressing *SIMKK RNAi* construct. The *SIMKK RNAi* line was selected on the basis of hairpin detections by PCR (Figures S1A,B, arrows) and subsequent *SIMKK* transcript quantitation by RT qPCR (Figure S1C) showing reduction of *SIMKK* transcript by 57% compared to the wild type RSY (Figure S1C). Leaf extracts of control *M. sativa* line RSY as well as extracts from *M. sativa SIMKK RNAi* line with knock downed *SIMKK* were probed with anti-pTEpY antibody before and after wounding. In this case, the anti-pTEpY antibody recognized two bands with the top one corresponding to phosphorylated SIMK (Figure 2A, top panel, top arrow, pSIMK) and the bottom one to an unknown wound-inducible MAPK species (Figure 2A, top panel, bottom arrow, question mark). The band corresponding to pSIMK was significantly reduced in the *SIMKK RNAi* line (Figure 2A, top panel, top arrow, pSIMK). When the same extracts were immunoblotted with anti-SIMK antibody to verify the position of SIMK, it was found that the total levels of SIMK were also reduced in the *SIMKK RNAi* line (Figure 2A, bottom panel, arrow, SIMK). In western blots following Zn^2+^/Phos-Tag™ gel of the same extracts as in Figure 2A, the control line showed the separation of two SIMK immunoreactive bands, one corresponding to the phosphorylated SIMK (Figure 2B, top arrow, pSIMK) while the bulk immunoreactivity resided in the lower band corresponding to non-phosphorylated SIMK (Figure 2B, bottom arrow). By contrast, extracts of *SIMKK RNAi* line tested hereby failed to show the upper band corresponding to phosphorylated SIMK (Figure 2B).

### Phosphorylation of monocot proteins

Next we used Phos-Tag™ electrophoretic separation to document phosphorylation of alpha-tubulin in hyperosmotically-treated *Hordeum vulgare* and *Triticum turgidum* seedlings as previously published for *Oryza sativa* and *Arabidopsis thaliana* (Ban et al., 2013; Fujita et al., 2013). In the case of *Hordeum vulgare*, the efficient separation and detection of hyperosmotically-induced protein phosphorylation was further validated in the case of barley MAPK (HvMPK4) detected with a custom raised antibody.

**Figure 3:**
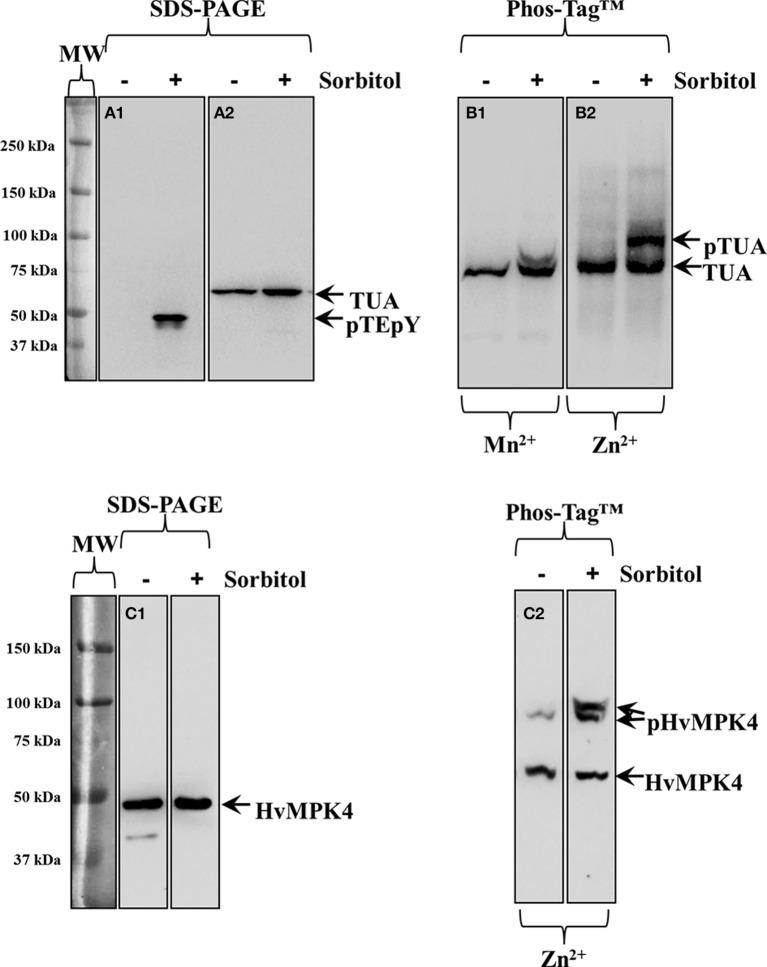
**Hyperosmotically induced (0.8 M sorbitol for 30 min) phosphorylation of alpha-tubulin and HvMPK4 as probed in barley root extracts. (A1)** Severe, non-ionic hyperosmotic stress induces activation of unknown MAPKs recognized by pTEpY antibody. **(A2)** Under the same conditions total levels of alpha-tubulin are slightly upregulated as evidenced in immunoblots following standard SDS-PAGE. **(B1)** Phos-Tag™ gel separates alpha-tubulin to a phosphorylated (top arrow, pTUA) and a non-phosphorylated band (bottom arrow TUA) in the presence of Mn^2+^. **(B2)** A wider separation of phosphorylated (top arrow, pTUA) from non-phosphorylated alpha tubulin (bottom arrow, TUA) is seen in the presence of Zn^2+^ in the Phos-Tag™ gel indicating better efficiency of Zn^2+^ containing Phos-Tag™ gels to separate phosphorylated tubulin. The panels on the left of **(A1)** corresponds to image of the protein ladder following Ponceau S staining of the membrane. **(C1)** Identical extracts as those used in Panel **(A)** where probed with anti-HvMPK4 antibody. The antibody specifically recognizes HvMPK4 (left arrow) and further shows conservation of total HvMPK4 levels in control and hyperosmotically-treated samples. **(C2)** Hyperosmotically-induced phosphorylation of HvMPK4 as monitored by Zn^2+^/Phos-Tag™ gel electrophoresis and western blot. The lower band corresponds to unphosphorylated HvMPK4 (bottom arrow) while the upper bands correspond to different phosphorylation statuses of HvMPK4 after hyperosmotic induction (right lane, top arrows, pHvMPK4). In untreated samples (left lane) only one faint upper band appears probably corresponding to basally phosphorylated HvMPK4. Panels on the left of **(A1)** and **(C1)** correspond to images of the protein ladder following Ponceau S staining of the membranes.

In western blots of barley extracts after sorbitol treatment, an anti-pTEpY antibody recognized one prominent band which was not apparent in extracts of resting plants (Figure 3A1). Alpha-tubulin immunoreactivity was slightly enhanced in extracts from hyperosmotically treated plants (Figure 3A2 right lane cf left lane). Phosphorylation of alpha-tubulin following hyperosmotic treatment was monitored in western blots following both Mn^2+^/ and Zn^2+^/Phos-Tag™ gel separation (Figures 3B1,B2 respectively). While in both cases, tubulin immunoreactivity appeared as two bands (right lanes of Figures 3B1,B2) compared to extracts of untreated samples (left lanes of Figures 3B1,B2), the separation of these two bands was clearly better in western blots following Zn^2+^/Phos-Tag™ gels highlighting the efficiency of Zn^2+^/Phos-Tag™ gels to separate phosphorylated (pTUA) from non-phosphorylated alpha-tubulin (TUA) compared to Mn^2+^/Phos-Tag™ gels. Further we studied, as a proof-of-principle, the hyperosmotically induced phosphorylation of a previously uncharacterized barley ortholog of *Arabidopsis thaliana* MPK4 (HvMPK4) using a custom raised antibody. The specificity of the antibody was corroborated in western blots following standard SDS-PAGE showing a band at ca. 43 kDa which remained roughly conserved between extracts of untreated and hyperosmotically treated plants (Figure 3C1 left and right panel respectively). By Zn^2+^/Phos-Tag™ analyses, it was found that the HvMPK4 immunoreactivity appeared in two bands in extracts of untreated plants, the lower of which corresponded to unphosphorylated HvMPK4 (Figure 3C2 left lane, bottom arrow, HvMPK4) while the upper one probably corresponded to basally phosphorylated HvMPK4 (Figure 3C2 left lane, top arrow, pHvMPK4). Interestingly, in extracts of hyperosmotically treated plants, the HvMPK4 immunoreactivity breaks down to three bands. Again the lowest corresponding to the non-phosphorylated HvMPK4 (Figure 3C2 right lane, bottom arrow, HvMPK4), while the two upper ones that reside close together probably reflected two different phosphorylation states of HvMPK4 induced after hyperosmotic treatment (Figure 3C2 right lane, top arrows, pHvMPK4).

**Figure 4:**
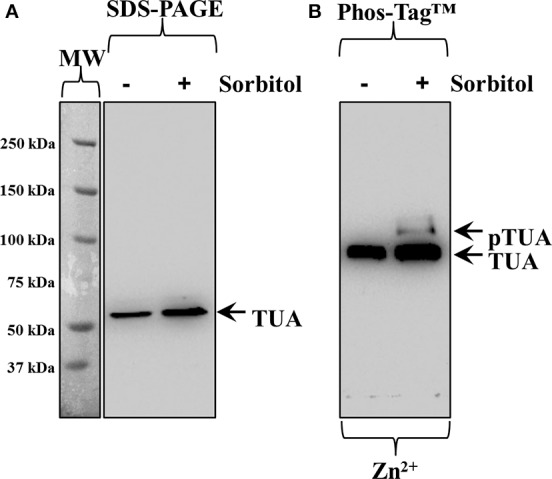
**Hyperosmotically induced (0.8 M sorbitol for 30 min) phosphorylation of alpha-tubulin as probed in wheat root extracts. (A)** Hyperosmotic stress induces small increase of alpha-tubulin (left arrow, TUA) levels as evidenced in standard SDS-PAGE followed by immunoblot analysis. **(B)** Zn^2+^/Phos-Tag™ analysis of the same extract, shows phosphorylation of a fraction of alpha-tubulin (top arrow, pTUA) compared to extracts of control, untreated roots. The bottom arrow denotes unphosphorylated alpha-tubulin. The panel left to **(A)** corresponds to image of the protein ladder following Ponceau S staining of the membrane.

Tubulin phosphorylation was also followed in extracts of hyperosmotically-treated wheat seedlings. By standard SDS-PAGE and subsequent western blot, alpha-tubulin immunoreactivity was detected as a single band, showing slight upregulation of alpha-tubulin intensity after the hyperosmotic treatment (Figure 4A, right lane cf left lane corresponding to control). As previously described for barley, Zn^2+^/Phos-Tag™ gels discriminated tubulin immunoreactivity in two bands, the upper one of which likely corresponded to phosphorylated alpha-tubulin (Figure 4B, top arrow, pTUA) while the lower one corresponded to non-phosphorylated alpha tubulin (Figure 4B, bottom arrow, TUA).

### Validation of the protocol with extracts of *A. thaliana*

When executing the protocol it is best to characterize proteins which are known to undergo conditional phosphorylation. In the case of *Arabidopsis thaliana* this is best exemplified in the case of the MPK6, representing mitogen activated protein kinase known to be activated by oxidative stress (Wang et al., 2013).

**Figure 5:**
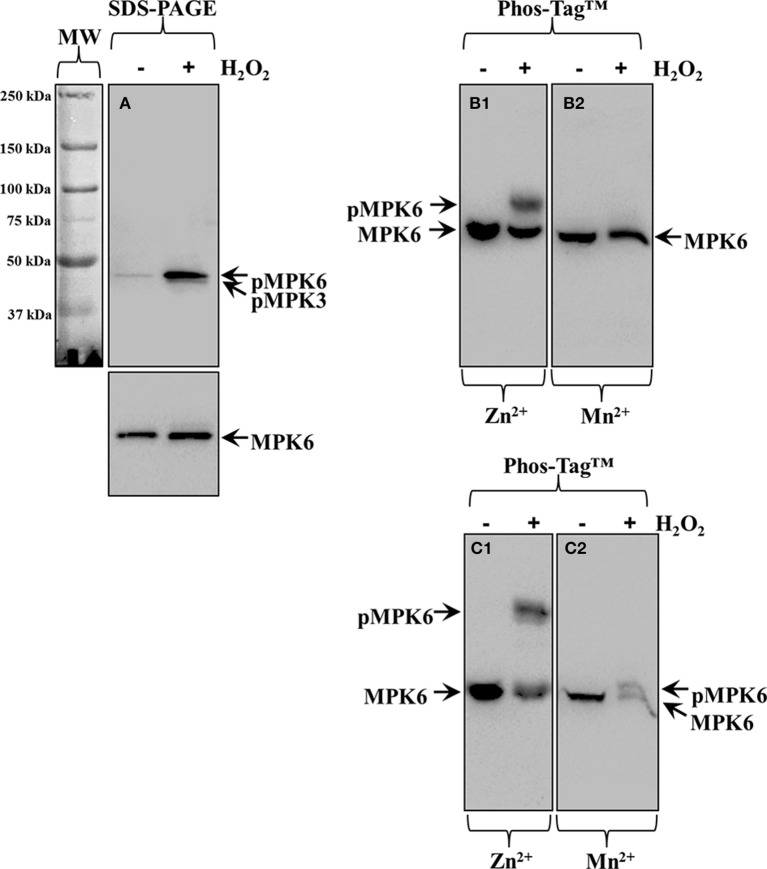
**Overview of the protocol applied on** ***Arabidopsis thaliana*** **root protein extracts following treatment with 15 mM H_2_O_2_ for 30 min. (A)** Immunoblot detection of MPK6 (bottom panel, arrow, MPK6), and phosphorylated MPK6 (top panel, top arrow, pMPK6) in conventional SDS-PAGE gels by means of anti-pTEpY antibody. In top panel of **(A)** the fainter band likely corresponds to phosphorylated MPK3 (bottom arrow, pMPK3) which is detectable depending on the separation efficiency of the gel. **(B1,B2)** Identical immunoblot from Phos-Tag™ separated samples in the presence of Zn^2+^ **(B1)** or Mn^2+^ **(B2)** immunodetected with antibody against MPK6 **(B1,B2)**. Proteins were allowed to separate in the Phos-Tag™ gel for 4 h. **(C1,C2)** Identical immunoblots from Phos-Tag™ separated samples in the presence of Zn^2+^ **(C1)** or Mn^2+^ **(C2)**. Proteins were allowed to separate for 6 h in the Phos-Tag™ gel. Panels on the left of **(A)** correspond to images of the protein ladder following Ponceau S staining of the membrane.

Oxidative stress, performed by treatment of *Arabidopsis thaliana* seedlings with 15 mM H_2_O_2_ for 30 min, is not inducing changes in total MPK6 (Figure 5A, bottom panel, arrow, MPK6) levels but does promote the accumulation of phosphorylated MAPK species probed with phosphospecific antibody (Figure 5A, top panel, top arrow, pMPK6). Depending on the separation efficiency of the gel, the phosphospecific antibody may detect two (Figure 5A, top panel, arrows) bands, the most prominent of which corresponds to MPK6 (Figure 5A, top panel, top arrow, pMPK6) while the lower probably corresponds to phosphorylated MPK3 (Figure 5A, top panel, bottom arrow, pMPK3; see also Willmann et al., 2014). Phos-Tag™ analyses show that the H_2_O_2_-induced accumulation of phosphorylated MPK6 can be discriminated by both the Mn^2+^ and the Zn^2+^ variants with variable efficiency and depending on the time of electrophoretic separation. The separation of dually-phosphorylated MPK6 (Figures 5B1,C1,C2, top arrows, pMPK6) from its non-phosphorylated counterpart (Figures 5B1,C1,C2, bottom arrows, MPK6) is depending on the transition metal used in Phos-Tag™ gel assembly (Figure 5B1 of Figure 5B2 showing better separation in the presence of Zn^2+^ compared to Mn^2+^) but also depends on gel running time allowing better separation after longer times of electrophoresis (Figures 5B1,B2 come from blots of Phos-Tag™ gels separated for 4 h while Figures 5C1,C2 come from gels separated for 6 h).

**Figure 6:**
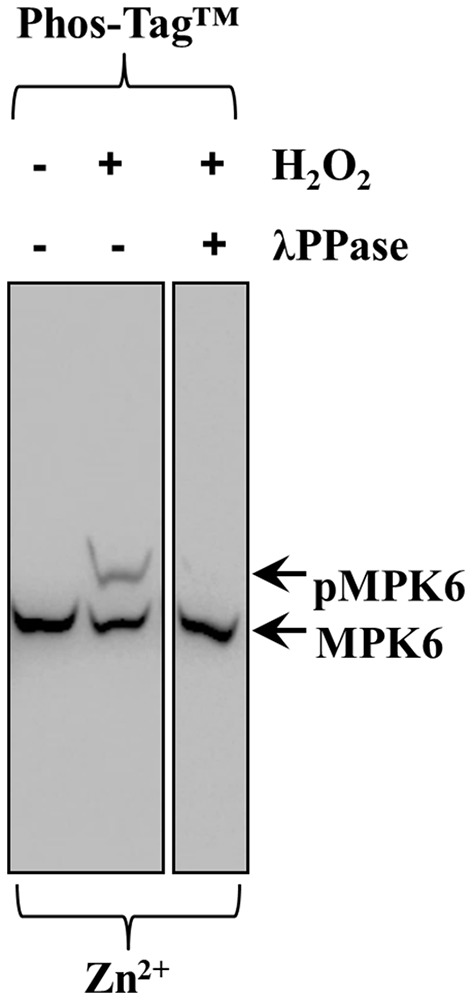
**Effects of λ PPase treatment on the ability of Zn^2+^/Phos-Tag™ gel to discriminate phosphorylated MPK6 (pMPK6) in root extracts of *Arabidopsis thaliana* seedlings treated with 15 mM H_2_O_2_ for 30 min**. Extracts untreated with λ PPase show an upper band corresponding to phosphorylated MPK6 (top arrow, pMPK6) and after treatment with λ PPase the upper band is eliminated. The lower band (arrow, MPK6) corresponds to the unphosphorylated MPK6.

In order to verify that Phos-Tag™ gels truly discriminate between phosphorylated and non-phosphorylated protein species a control experiment should be carried out. In the case of H_2_O_2_-induced phosphorylation of MPK6, we eliminated MPK6 phosphorylation (Figure 6 middle lane, top arrow, pMPK6) by λ PPase treatment of the protein extract, leading to the disappearance of phosphorylated MPK6 in the respective Phos-Tag™ gel (Figure 6) allowing the detection of non-phosphorylated MPK6 alone (last lane, bottom arrow, MPK6).

## Discussion

### Development of the protocol

There are two principal strategies for implementing acrylamide pendant Phos-Tag™ separation of phosphorylated proteins from their non-phosphorylated counterparts and these differ on the transition metal cation used. The original method utilized Mn^2+^ on a standard Tris-based SDS PAGE (Kinoshita et al., 2006) and in our earlier studies we used it as such (Beck et al., 2010; Panteris et al., 2010). Later, improved electrophoretic transfer of Phos-Tag™ separated proteins of variable M*_r_* was reported using Zn^2+^ instead, incorporated to a NuPAGE setup with Bis-Tris based gels and MOPS-Tris-based electrophoresis buffer (Kinoshita and Kinoshita-Kikuta, 2011; Kinoshita et al., 2012; Kinoshita-Kikuta et al., 2014), which we appropriately adopted and modified for the separation of phosphorylated proteins from whole extracts of *Arabidopsis thaliana* seedlings or roots (Komis et al., 2014; Smékalová et al., 2014).

### Importance of divalent cation in acrylamide pendant Phos-Tag™ separation of phosphorylated proteins

We found that both Phos-Tag™ variants are useful for the separation of phosphorylated proteins and their non-phosphorylated counterparts, provided careful management of electrophoresis running time and thorough post-treatment of the Phos-Tag™ gel to ensure quantitative protein transfer to PVDF membrane. Thus, as we showed in the case of H_2_O_2_-induced phosphorylation of *Arabidopsis thaliana* MPK6, the separation of the phospho- from the non-phosphorylated form is more efficient in the Zn^2+^/Phos-Tag™ regime and after a gel running time of 6 h instead of 4 h (Figures 5B1,C1). Ultimately, the running time will depend on the size of the protein of interest, but for smaller proteins than those detected herein, higher acrylamide percentages may be used to prevent the elution of such proteins from the gel during prolonged electrophoresis.

### Importance of the extraction buffer in the preservation of phosphorylated groups

Preservation of phosphorylated groups in crude protein extracts is of paramount importance for the successful detection of phosphorylated proteins by Phos-Tag™, since such proteins frequently occur as a minute fraction of the total protein pool. Therefore, it was crucial to establish extraction conditions to preserve phosphorylation status of the proteins under study. From three common extraction buffers used for protein isolation, we found that buffer E and especially buffer B, were superior to RIPA buffer. We believe that the presence of phosphates in buffer B, further prevents spontaneous protein dephosphorylation which might occur during prolonged storage of protein extracts, even in the presence of phosphatase inhibitors.

## Importance of post-electrophoretic processing of the gel

Phosphorylated proteins remain bound in Phos-Tag™ gels by transition metal bridges. In order to ensure their quantitative transfer to solid supports like PVDF membranes, divalent cations have to be efficiently removed. This is normally done by washing gels in solutions of chelating agents such as EDTA. The original protocol for post-electrophoretic treatment of Phos-Tag™ gels suggested 10 min incubation time in transfer buffer containing 1 mM EDTA (Kinoshita et al., 2006). We and others (Ban et al., 2013; Smékalová et al., 2014), found this step insufficient for quantitative transfer of proteins from the Phos-Tag™ gel to the PVDF membrane and thus we increased the concentration of EDTA to 10 mM while increasing the incubation time to 1 h with as many as six changes of the washing solution. It is critical, however, to omit methanol from washing steps as it may fix proteins to the gel and prolong the washing as much as possible and with as many changes as possible until protein diffusion might become a problem.

### Applications of the method

We have used acrylamide pendant Phos-Tag™ to delineate the phosphorylation status of the microtubule associated protein 65-1 (MAP65-1) in four MAPK-related mutants (*anp2 × anp3, mpk4, yda*, and *ΔNyda*) of *Arabidopsis thaliana* and also showed phosphorylation of *At*MPK4 under conditions of hyperosmotic stress in wild type *Arabidopsis thaliana* seedlings (Beck et al., 2010; Smékalová et al., 2014). Moreover, we showed phosphorylation of MAP65-1 ortholog of *Vigna sinensis* following hyperosmotic stress (Panteris et al., 2010).

In the plant science, acrylamide pendant Phos-Tag™ had been further used to demonstrate alpha-tubulin phosphorylation in *Arabidopsis thaliana* and rice following hyperosmotic and salt stress or antimicrotubular drug treatments associated with the function of an atypical kinase originally identified as phosphatase (Ban et al., 2013; Fujita et al., 2013). Phos-Tag™ was also used to follow the pathogen elicited phosphorylation of either plant or pathogen proteins during the development of innate immune responses (Mao et al., 2011; Taylor et al., 2012; Li et al., 2014) and the MPK6-mediated phosphorylation of ethylene responsive factor ERF104 (Bethke et al., 2009) and the BES1 (BRI1-EMS suppressor 1) transcription factor (Kang et al., 2015) among others.

Herein we provide a survey of acrylamide pendant Phos-Tag™ extending its application to discriminate phosphorylated from non-phosphorylated protein species in two monocots (barley and wheat) and one dicot crop (alfalfa). By means of Zn^2+^/Phos-Tag™ we showed the hyperosmotically induced phosphorylation of alpha-tubulin as previously described for rice and *Arabidopsis thaliana* (Ban et al., 2013; Fujita et al., 2013). It is likely that tubulin phosphorylation detected herein, corresponds to Thr-349 as previously reported (Ban et al., 2013). However, α-tubulin might be also targeted for Tyr phosphorylation regulating microtubule sensitivity to low temperatures (Sheremet et al., 2012a), growth and morphogenesis of Arabidopsis root cells (Yemets et al., 2008) and mitotic progression in dividing tobacco BY-2 suspension culture cells (Sheremet et al., 2012b). Although, the identity of phosphorylated residues in tubulin (and any other protein) cannot be revealed by Phos-Tag™, the method can be readily combined with immunoprecipitation of the protein of interest and subsequent probing of the resulting western blot with phospho-amino acid specific antibodies. Moreover, we demonstrated the hyperosmotically induced accumulation of phosphorylated HvMPK4 in appropriately treated barley plants. It was of interest to see that by the use of an otherwise monospecific custom raised antibody, the phosphorylated form of HvMPK4 was further discriminated in two bands. During MAPK activation the TXY motif within the activation loop becomes simultaneously dually phosphorylated and indeed in the case of *Arabidopsis thaliana* MAPKs (e.g., MPK4 and MPK6) separated by Phos-Tag™ we only detected one upper band corresponding to pMPK6 (Beck et al., 2010; Smékalová et al., 2014). The appearance of two upper bands probably reflects to two different phosphorylation states of HvMPK4, one probably corresponding to the dually phosphorylated TEY motif while the second one might correspond to another phosphorylation site that will require further attention.

On the other hand Phos-Tag™ helped to corroborate the previously published robust reaction of *Medicago sativa* SIMK in root extracts exposed to H_2_O_2_, salt and hyperosmotic stress (Kiegerl et al., 2000; Ovečka et al., 2014) without a phospho-specific antibody. Additionally, we aimed to extend the application of Phos-Tag™ in order to characterize a transgenic *Medicago sativa* line with downregulated SIMKK, an upstream regulator of SIMK (Cardinale et al., 2002; Ovečka et al., 2014), by using siRNA approach. The SIMKK RNAi line used herein showed decreased accumulation of pSIMK in western blots after standard SDS-PAGE probed with anti-pTEpY antibody and no separation of pSIMK from SIMK following Zn^2+^/Phos-Tag™. The reduced anti-pTEpY immunoreactivity of SIMK and the absence of a band corresponding to pSIMK from western blot that followed Zn^2+^/Phos-Tag™, may be explained by the reduced levels of SIMK upstream regulator, SIMKK (Figure S1B) and the severe downregulation of endogenous SIMK in the RNAi line even if equal loading was verified by Ponceau S staining. This rather unexpected finding will merit further attention.

### Comparison with other methods

Acrylamide pendant Phos-Tag™ identification and monitoring of protein phosphorylation is a small scale method and thus will be compared to similar approaches. The identification of phosphorylated protein species and their subsequent quantitative analysis requires their efficient separation from their non-phosphorylated counterparts. In small scale experiments, phosphorylation can be monitored by metabolic labeling of phosphorylated proteins with radioactive ATP (γ^32^P-ATP; Cuenda, 2000; Dickson, 2008). Radioactive phosphorus is a beta emitter and exhibits the highest emission energy compared to other biologically used radioisotopes. For this reason γ^32^P-ATP handling requires dedicated laboratory space, equipment and special training of the personnel, adhering to institutional rules for storage, handling and disposal of radioactive waste. Although γ^32^P-ATP comes at affordable prices by many vendors, the health risks and the cost for establishing appropriate laboratory space and training of handling personnel make the use of γ^32^P-ATP prohibitive especially for research groups that only occasionally study protein phosphorylation.

Alternatively, protein phosphorylation may be semi quantitatively addressed on standard immunoblots following denaturing SDS PAGE with phosphorylation specific antibodies (Willmann et al., 2014). Although some commonly used phospho-specific antibodies are commercially available most will have to be custom ordered at a significant cost.

A last important alternative to Phos-Tag is the one dimensional electrophoretic separation of phosphorylated proteins by means of isoelectric focusing coupled to western blot analysis (Anderson and Peck, 2008, 2014). Although there are no principal differences with Phos-Tag™ one must take into account the necessity of specialized chemicals, high voltage power supplies and a setup which is more complicated and expensive than the acrylamide pendant Phos-Tag approach presented hereby.

### Conflict of interest statement

The authors declare that the research was conducted in the absence of any commercial or financial relationships that could be construed as a potential conflict of interest.
