# Supplementary material for: Monitoring protein phosphorylation by acrylamide pendant Phos-Tag™ in various plants
Source: Front Plant Sci. 2015 May 13;6:336. doi: 10.3389/fpls.2015.00336 (PMC4429547; doi:10.3389/fpls.2015.00336)
Supplement: Supplementary file 1 [file Image1.PDF]

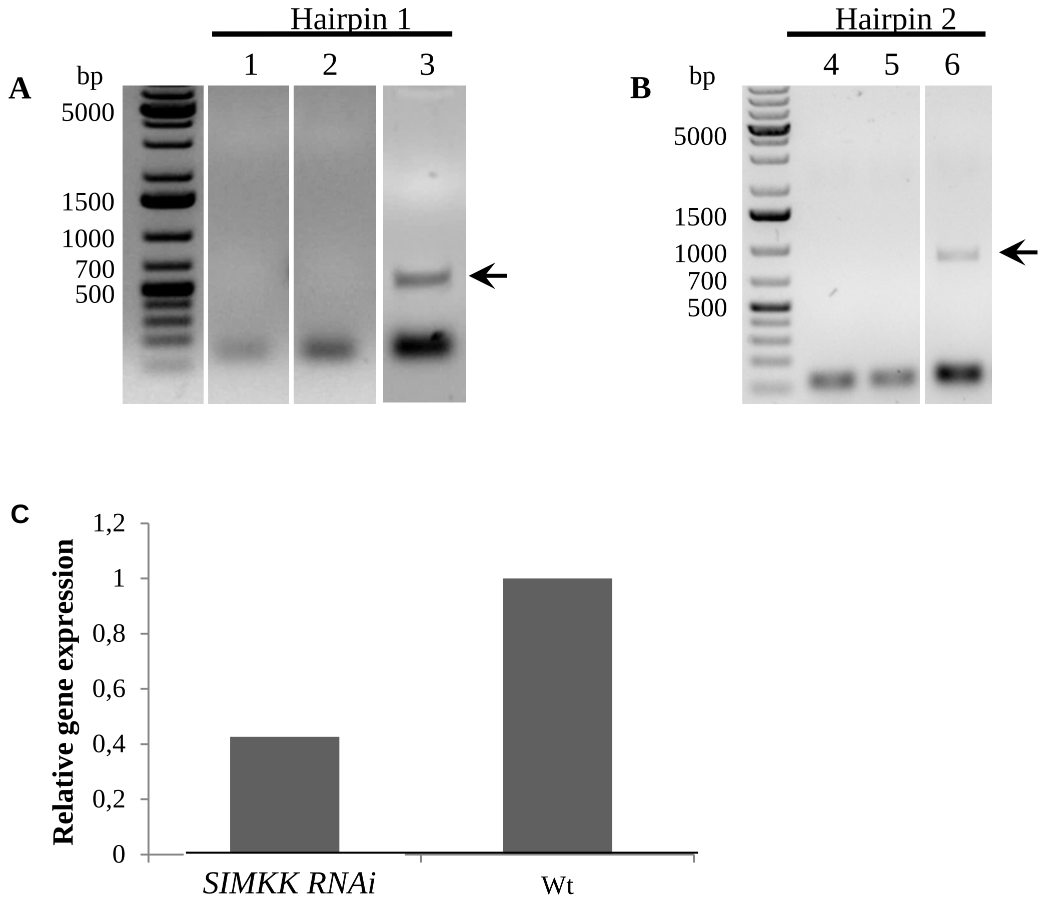

Figure S1. Validation and characterization of *SIMKK RNAi* line of *M. sativa*.

(A) PCR results on the detection of hairpin 1 (arrow) in *SIMKK RNAi* line of *M. sativa*. Lane description 1 - Negative control (H_2O), 2 - Negative control (wild type *Medicago sativa* cv. RSY), 3 - *SIMKK RNAi* line of *Medicago sativa* (528 bp band indicates presence of hairpin 1).

(B) PCR results on the detection of hairpin 2 (arrow) in *SIMKK RNAi* line of *M. sativa*. Lane description: 4 - Negative control (H_2O), 5 - Negative control (wild type *Medicago sativa* cv. RSY), 6 - *SIMKK RNAi* line of *Medicago sativa* (814 bp band indicates presence of hairpin 2)

(C) Suppression of the *SIMKK* transcripts in *Medicago sativa* cv. RSY in plant harboring *SIMKK RNAi* construct as quantified by RT qPCR. The expression level of *SIMKK* in the transgenic line was normalized to the expression level of *ACTIN2* gene and it is shown as relative to the expression of *SIMKK* in control plant.
